# Bone microarchitecture and strength in obese women with and without polycystic ovary syndrome (PCOS): a peripheral quantitative computed tomography (pQCT) study

**DOI:** 10.3389/fendo.2026.1935187

**Published:** 2026-09-25

**Authors:** Yuxing Zhao, Qiuying Zhang, Fengying Gong, Lian Duan, Yuelun Zhang, Linjie Wang, Huijuan Zhu, Weibo Xia

**Affiliations:** 1 Key Laboratory of Endocrinology of National Health Commission, Department of Endocrinology, State Key Laboratory of Complex Severe and Rare Diseases, Peking Union Medical College Hospital, Chinese Academy of Medical Science and Peking Union Medical College, Beijing, China; 2 Chinese Academy of Medical Sciences and Peking Union Medical College, Beijing, China; 3 Medical Research Center, Peking Union Medical College Hospital, Chinese Academy of Medical Sciences and Peking Union Medical College, Beijing, China

**Keywords:** bone microarchitecture, HR-pQCT, obesity, PCOS, testosterone

## Abstract

**Background:**

Polycystic ovary syndrome (PCOS) is a prevalent endocrine disorder, but its skeletal impact remains unclear due to confounding obesity. Areal bone mineral density (BMD) studies are conflicting, with body mass index (BMI) as a modifier. High-resolution peripheral quantitative computed tomography (HR-pQCT) data in PCOS are scarce, and no study has compared obese PCOS women to BMI-matched controls or tested hyperandrogenemia as a mediator.

**Methods:**

This cross-sectional study enrolled 23 obese women with PCOS (BMI ≥28 kg/m²), 21 BMI-matched obese women without PCOS, and 24 normal-weight controls. PCOS was diagnosed using Rotterdam criteria. Participants underwent anthropometric measurements, biochemical assays, and HR-pQCT scanning of distal radius and tibia with finite element analysis. Group comparisons employed ANOVA or Kruskal-Wallis tests with selective Bonferroni correction. Linear regression examined associations between bone parameters and metabolic/inflammatory markers, adjusting for age and BMI. Mediation analysis tested whether testosterone mediated group differences in bone parameters.

**Results:**

Compared with the BMI-matched obese (OB) group, the OB-PCOS group exhibited higher total volumetric BMD (vBMD) at both the radius (p = 0.036) and tibia (p = 0.018), and higher cortical vBMD (radius: p = 0.008; tibia: p = 0.017). At the tibia, OB-PCOS additionally showed higher trabecular vBMD (p = 0.008) and trabecular bone volume fraction (p = 0.008). Despite these density advantages, no significant differences in estimated bone strength (stiffness and failure load) were observed between the two obese groups at either skeletal site (all p > 0.05). Higher BMI was associated with improved trabecular microarchitecture at the radius and increased estimated bone strength at the tibia. No mediating effect of testosterone was found for the bone differences associated with PCOS.

**Conclusions:**

PCOS is associated with increased volumetric BMD independent of obesity; however, this advantage does not translate into enhanced estimated bone strength, suggesting alterations in bone material properties. No evidence was found that testosterone mediates these skeletal differences, implicating alternative PCOS-related mechanisms (e.g., chronic inflammation or insulin resistance) in their etiology. These findings highlight the complex interplay between PCOS and obesity in skeletal health and underscore the need for longitudinal studies to assess fracture risk in this population.

## 1 Introduction

Polycystic ovary syndrome (PCOS) is one of the most common endocrine disorders affecting women of reproductive age, with a prevalence ranging from 6% to 20% depending on the diagnostic criteria employed (1, 2). Characterized by hyperandrogenism, ovulatory dysfunction, and polycystic ovarian morphology, PCOS is frequently accompanied by metabolic disturbances, including insulin resistance, hyperinsulinemia, and obesity (2, 3). Very recently, a new nomenclature—Polyendocrine Metabolic Ovarian Syndrome (PMOS)—has been proposed to better capture the metabolic dimension of this condition (4). Given the established roles of reproductive hormones in skeletal homeostasis, particularly androgens and estrogens (2, 5), it is plausible that the hormonal and metabolic alterations inherent to PCOS may significantly impact bone health. However, the direction of this impact remains a subject of ongoing debate, largely due to the confounding effects of obesity, which frequently coexists with PCOS.

Previous studies investigating bone mineral density (BMD) in women with PCOS have yielded conflicting results. Some reports suggest that PCOS is associated with increased BMD, potentially due to the anabolic effects of hyperandrogenism on bone (6–8),while others have demonstrated reduced BMD or no significant differences compared to controls (2, 9, 10). A recent systematic review and meta-analysis attempted to reconcile these discrepancies by stratifying according to body mass index (BMI) (11). The results demonstrated distinct skeletal patterns: participants with PCOS and a BMI below 27 kg/m² exhibited decreased BMD at both vertebral and non-vertebral sites, along with reduced markers of bone formation and elevated markers of bone resorption. In contrast, those with PCOS and a BMI exceeding 27 kg/m² displayed higher BMD, while markers of bone turnover remained largely unchanged (11). This suggests that BMI may be a critical modifier of the skeletal phenotype in PCOS and that obesity may confer a protective effect on bone mass in this population.

Obesity itself exerts complex and site-specific effects on bone. It has long been recognized, through dual-energy X-ray absorptiometry (DXA) measurements, that obesity is associated with higher areal bone mineral density (12). More recently, the application of high-resolution peripheral quantitative computed tomography (HR-pQCT) has significantly advanced our understanding of bone microarchitecture in this population (13). Obese adults have been shown to exhibit thicker cortices, higher trabecular BMD and trabecular number, and lower cortical porosity compared to normal-weight adults, particularly at weight-bearing sites (13, 14). However, these favorable microarchitectural features may not be proportionate to the increased mechanical load, and the relationship between obesity and fracture risk appears to vary by skeletal site (15, 16). Furthermore, beyond simple body weight or BMI, appendicular lean mass has emerged as a key determinant of bone parameters, and has been linked to significant improvements in bone geometry and microarchitecture in obese postmenopausal women (15).

Despite these advances, important knowledge gaps remain. First, the application of HR-pQCT to assess bone health in PCOS remains limited, as most studies have relied on DXA-derived areal BMD, which is susceptible to confounding by body size and adiposity (17). HR-pQCT offers the advantage of three-dimensional assessment of volumetric BMD, cortical and trabecular microarchitecture, and estimated bone strength derived from finite element analysis (18). Collectively, these parameters provide a more comprehensive reflection of bone quality. Second, to our knowledge, no study has been published that directly compares bone microarchitecture between obese women with PCOS and BMI-matched obese women without PCOS, thereby isolating the effect of PCOS itself from that of adiposity. Third, hyperandrogenemia, a hallmark of PCOS, has not been systematically examined for its potential mediating role in explaining group differences in bone parameters (6). The present study was designed to address these three knowledge gaps.

Therefore, this study aimed to: (1) compare bone microarchitecture and estimated bone strength, assessed by HR-pQCT, between obese women with PCOS and BMI-matched obese women without PCOS; (2) explore the correlations between serum metabolic/inflammatory markers and bone parameters in the context of obesity with or without PCOS, and investigate whether serum testosterone mediates the observed differences in bone parameters between these two groups; and (3) compare bone parameters between each obese group (obese women with PCOS and obese women without PCOS) and normal-weight healthy controls to elucidate the independent effects of obesity and PCOS. We hypothesized that PCOS would exert distinct effects on bone microarchitecture independent of obesity, and that these effects would not be fully explained by differences in circulating testosterone levels.

## 2 Methods

### 2.1 Subjects

This study included three groups of adult women: obese women with PCOS (OB-PCOS group), obese women without PCOS (OB group), and normal-weight controls (CON group). For the OB-PCOS group, 23 obese women with PCOS (BMI ≥ 28 kg/m²) were consecutively enrolled. These participants attended the endocrinology outpatient clinic at Peking Union Medical College Hospital (PUMCH) and had undergone at least one HR-pQCT scan between 2019 and 2023. For comparison, 21 obese women without PCOS (BMI ≥ 28 kg/m²) were selected as the OB group. These participants were matched to the OB-PCOS group for age and BMI at the group level. Additionally, 24 healthy, normal-weight women (BMI < 28 kg/m²) were recruited as controls (CON group), with no significant age difference across the three groups (**Supplementary Figure S1**). In this study, the primary comparison of interest was between the two obese groups (OB-PCOS vs. OB), as this design allowed us to isolate the effect of PCOS on bone parameters independent of obesity. The normal-weight CON group was included for auxiliary purposes, as to provide a reference frame for interpreting the direction and magnitude of skeletal alterations in obese individuals. PCOS was diagnosed according to the Rotterdam criteria after exclusion of related disorders (19, 20), requiring the presence of at least two of the following three features: (1) clinical and/or biochemical hyperandrogenism; (2) ovulatory dysfunction (e.g., menstrual disorders, oligomenorrhea, or amenorrhea); and (3) polycystic ovarian morphology.

The inclusion criteria for all participants were as follows: (1) female sex; (2) aged 18 to 60 years; and (3) availability of HR-pQCT data for the radius and/or tibia. The exclusion criteria were: (1) presence of other metabolic bone diseases (e.g., Paget’s disease, osteomalacia, hyperparathyroidism); (2) use of medications known to significantly affect bone metabolism within the previous six months, including anti-osteoporotic drugs, glucocorticoids, and oral contraceptives or other estrogen-progestin preparations (e.g., Diane-35); (3) presence of any acute or active comorbid condition, including but not limited to active infection, recent major trauma or surgery, acute hepatic or renal failure, active autoimmune disease, or advanced malignant tumors; and (4) perimenopausal or postmenopausal status. Menopause was defined as 12 consecutive months of spontaneous amenorrhea or a history of bilateral oophorectomy. In women with PCOS-related oligomenorrhea or amenorrhea, premenopausal status was determined by clinical history and, when necessary, serum FSH levels, rather than by menstrual regularity alone.

### 2.2 Anthropometric data collection

Anthropometric assessments were conducted for all participants. Standing height was measured using a digital stadiometer (Hampton, Seritex, Inc., USA), and body weight was recorded using an electronic scale (RGZ-120, Xiheng, China). Measurements were taken to the nearest 0.1 cm and 0.1 kg, respectively. BMI was calculated as weight in kilograms divided by height in meters squared (kg/m²).

### 2.3 Biochemical assays

Fasting venous blood samples were collected from all participants. Routine laboratory measurements, including complete blood count, comprehensive metabolic panel, sex hormones, and inflammatory markers, were performed at the PUMCH Clinical Laboratory according to standardized protocols. Detailed information regarding the specific biochemical assays and kit manufacturers is provided in **Supplementary Table S1** (see **Supplementary Appendix**).

### 2.4 Morphological and biomechanical analysis

#### 2.4.1 Image quantification

HR-pQCT evaluations were performed at the non-dominant distal radius and the ipsilateral distal tibia using an XtremeCT II scanner (Scanco Medical, Switzerland). Scans were performed at the intended site only if no prior fracture had occurred at that location (21). All *in vivo* scans were acquired following the manufacturer’s standard protocol, with the scanner operating at 60 kVp and 1000 μA, and an integration time of 100 ms. The scanning procedure began with a scout view to position the reference line at the respective endplate and define the measurement region. Subsequently, 110 contiguous slices were obtained proximal to the reference line, covering a 10.2 mm segment starting at 9.0 mm (radius) or 22.5 mm (tibia) from the reference line, with an isotropic voxel size of 82 μm (22). Prior to each scanning session, the scanner was calibrated using a phantom containing hydroxyapatite (HA) rods of five known densities (0, 100, 200, 400, and 800 mg HA/cm³) embedded in a soft-tissue-equivalent resin (QRM, Moehrendorf, Germany), and attenuation data were converted to equivalent HA densities using a linear calibration curve. Daily quality control was performed using the same phantom, with measurement error maintained within ±8 mg HA/cm³ (1% of the 800 mg HA/cm³ phantom value) and maximum RMS-CV ≤ 2% for all morphological parameters. All acquired images were systematically assessed for motion artifacts and assigned a quality grade on a scale from 1 to 5 (with 1 indicating no motion artifacts); images with a grade of 3 or higher were excluded from subsequent analysis (23).

#### 2.4.2 Finite element analysis

Morphological analysis of the scans was conducted following the methodology established by Boutroy et al. (22) to derive the following parameters of bone microarchitecture: total area (Tt.Ar), cortical area (Ct.Ar), and trabecular area (Tb.Ar); total volumetric bone mineral density (Tt.vBMD), cortical volumetric bone mineral density (Ct.vBMD), and trabecular volumetric bone mineral density (Tb.vBMD); trabecular number (Tb.N), trabecular separation (Tb.Sp), trabecular thickness (Tb.Th), trabecular network inhomogeneity (Tb.1/N.SD); and bone volume fraction (Tb.BV/TV). An automated segmentation algorithm (Image Processing Language, v5.08b, Scanco Medical) was used to quantify cortical porosity (Ct.Po), cortical thickness (Ct.Th), cortical perimeter (Ct.Pm), and cortical pore diameter (Ct.Po.Dm). For biomechanical assessment, finite element analysis was performed by converting the HR-pQCT images into mesh models using FAIM software (v6.0, Numerics88, Canada) to estimate bone stiffness (N/mm) (24). Bone failure load (N) was predicted using the Pistoia criterion (25).

### 2.5 Statistical analysis

#### 2.5.1 General approach

All statistical analyses were performed using R software (version 4.2.3; R Foundation for Statistical Computing, Vienna, Austria) (26). The threshold for statistical significance was set at a two-sided alpha level of 0.05. Prior to the main analyses, potential outliers were identified and excluded using the interquartile range (IQR) method. Data points that fell below first quartile (Q1) − 1.5 × IQR or above third quartile (Q3) + 1.5 × IQR were defined as outliers and removed from the dataset.

#### 2.5.2 Group comparisons

The primary analyses focused on the comparison between the two obese groups (OB-PCOS vs. OB), while comparisons involving the CON group were performed as auxiliary descriptive analyses. The normality of the distribution for continuous variables was assessed using the Shapiro-Wilk test, and the homogeneity of variances was verified by Levene’s test (27). For comparisons between two groups, the selection of an appropriate statistical test was guided by the outcomes of these assumption checks. Specifically, an independent samples t-test was used when both assumptions were met. When the normality assumption was satisfied but the assumption of equal variances was violated, Welch’s t-test was applied. For data that did not follow a normal distribution, the non-parametric Mann-Whitney U test was employed. For comparisons involving more than two groups, one-way ANOVA was used when assumptions were met; otherwise, the Kruskal-Wallis test was utilized. In cases where the Kruskal-Wallis test yielded a statistically significant result, Dunn’s *post hoc* test was subsequently conducted for pairwise comparisons. Continuous data are presented as mean ± standard deviation or median (interquartile range), as appropriate. To account for multiple comparisons, the p-values were adjusted using the Bonferroni correction (28). For multiple comparisons, Bonferroni correction was applied selectively. Specifically, for the primary confirmatory comparisons between the two obese groups (**Tables 1**, **2**), we report unadjusted p-values because these analyses were hypothesis-driven and designed to test specific *a priori* questions, and Bonferroni correction would be overly conservative given the interrelated nature of HR-pQCT parameters. For the auxiliary descriptive comparisons involving the CON group (**Table 3**), we applied Bonferroni correction to reduce the risk of false-positive findings in these exploratory analyses, and we explicitly flag these as descriptive rather than confirmatory. All adjusted p-values are clearly indicated in the tables with the notation “adj_p”.

**Table 1:** HR-pQCT parameters of the distal radius in OB-PCOS and OB groups.

HR-pQCT parameters	OB-PCOS group (n=23)	OB group (n=21)	p-value
Tt.Ar (mm2)	229.261 ± 40.167	248.690 ± 26.462	0.068
Tb.Ar (mm2)	171.683 ± 39.378	193.205 ± 29.050	0.047*
Ct.Ar (mm2)	61.435 ± 7.661	60.243 ± 7.572	0.607
Tt.vBMD (mg HA/cm3)	380.513 ± 54.465	342.171 ± 62.805	0.036*
Tb.vBMD (mg HA/cm3)	169.743 ± 30.688	159.300 ± 43.331	0.358
Ct.vBMD (mg HA/cm3)	955.757 ± 43.695	926.757 ± 38.987	0.008*
Tb.BV/TV	0.245 ± 0.043	0.232 ± 0.062	0.408
Tb.N (1/mm)	1.637 ± 0.224	1.558 ± 0.238	0.263
Tb.Th (mm)	0.224 ± 0.013	0.222 ± 0.015	0.785
Tb.Sp (mm)	0.579 ± 0.091	0.620 ± 0.119	0.209
Tb.1/N.SD (mm)	0.216 ± 0.042	0.235 ± 0.060	0.307
Ct.Pm (mm)	62.600 ± 6.323	66.038 ± 3.802	0.037*
Ct.Po (%)	0.003 ± 0.004	0.004 ± 0.002	0.037*
Ct.Th (mm)	1.164 ± 0.159	1.086 ± 0.176	0.067
Ct.Po.Dm (mm)	0.138 ± 0.020	0.153 ± 0.023	0.023*
Bone stiffness (N/mm)	65954.578 ± 10106.477	63859.952 ± 11530.358	0.524
Bone failure load (N)	3689.087 ± 568.287	3505.021 ± 635.391	0.316

Data are presented as mean ± SD.

Tt.Ar, total area; Tb.Ar, trabecular area; Ct.Ar, cortical area; Tt.vBMD, total volumetric bone mineral density; Tb.vBMD, trabecular volumetric bone mineral density; Ct.vBMD, cortical vBMD; Tb.N, trabecular number; Tb.Th, trabecular thickness; Tb.Sp, trabecular separation; Tb.1/N.SD, inhomogeneity of trabecular network; Tb.BV/TV, trabecular bone volume fraction (bone volume/tissue volume); Ct.Pm, cortical perimeter; Ct.Th, cortical thickness; Ct.Po, cortical porosity; Ct.Po.DM, cortical pore diameter.

Unadjusted p-values are reported for these primary confirmatory comparisons. Bonferroni correction was not applied due to the high intercorrelation among HR-pQCT parameters and to avoid type II error; see Methods for details.

For non-normally distributed variables, p-values were calculated using the Mann-Whitney U test.

*p < 0.05.

**Table 2:** HR-pQCT parameters of the distal tibia in OB-PCOS and OB groups.

HR-pQCT parameters	OB-PCOS group (n=12)	OB group (n=9)	p-value
Tt.Ar (mm2)	628.725 ± 68.847	690.456 ± 118.191	0.148
Tb.Ar (mm2)	503.367 ± 65.823	569.944 ± 120.312	0.120
Ct.Ar (mm2)	130.400 ± 16.705	125.833 ± 18.137	0.557
Tt.vBMD (mg HA/cm3)	347.575 ± 36.412	300.044 ± 48.025	0.018*
Tb.vBMD (mg HA/cm3)	186.967 ± 22.081	158.722 ± 21.426	0.008*
Ct.vBMD (mg HA/cm3)	968.717 ± 26.908	932.178 ± 37.145	0.017*
Tb.BV/TV	0.280 ± 0.028	0.242 ± 0.028	0.008*
Tb.N (1/mm)	1.458 ± 0.222	1.309 ± 0.224	0.129
Tb.Th (mm)	0.248 ± 0.011	0.247 ± 0.026	0.240
Tb.Sp (mm)	0.657 ± 0.099	0.760 ± 0.127	0.050
Tb.1/N.SD (mm)	0.261 ± 0.044	0.327 ± 0.070	0.015*
Ct.Pm (mm)	96.967 ± 5.483	102.100 ± 8.300	0.104
Ct.Po (%)	0.013 ± 0.005	0.016 ± 0.006	0.354
Ct.Th (mm)	1.558 ± 0.192	1.444 ± 0.236	0.234
Ct.Po.DM (mm)	0.208 ± 0.037	0.220 ± 0.029	0.457
Bone stiffness (N/mm)	204437.275 ± 30526.772	192614.850 ± 31433.487	0.383
Bone failure load (N)	11112.955 ± 1594.392	10518.231 ± 1625.399	0.398

Data are presented as mean ± SD.

Tt.Ar, total area; Tb.Ar, trabecular area; Ct.Ar, cortical area; Tt.vBMD, total volumetric bone mineral density; Tb.vBMD, trabecular volumetric bone mineral density; Ct.vBMD, cortical vBMD; Tb.N, trabecular number; Tb.Th, trabecular thickness; Tb.Sp, trabecular separation; Tb.1/N.SD, inhomogeneity of trabecular network; Tb.BV/TV, trabecular bone volume fraction (bone volume/tissue volume); Ct.Pm, cortical perimeter; Ct.Th, cortical thickness; Ct.Po, cortical porosity; CT.Po.DM, cortical pore diameter.

Unadjusted p-values are reported for these primary confirmatory comparisons. Bonferroni correction was not applied due to the high intercorrelation among HR-pQCT parameters and to avoid type II error; see Methods for details.

For non-normally distributed variables, p-values were calculated using the Mann-Whitney U test.

*: p < 0.05.

**Table 3:** Comparison of bone microarchitecture and estimated strength between OB, OB-PCOS and CON group.

Parameter	CON group (n=24)	OB group (n=21)	p_value (OB vs. CON)	adj_p (OB vs. CON)	OB-PCOS group (n=23)	p_value (OB-PCOS vs. CON)	adj_p (OB-PCOS vs. CON)
R_Ct.Ar (mm²)	57.733 ± 7.076	60.243 ± 7.572	0.322#	0.967#	61.435 ± 7.661	0.128	0.384
T_Ct.Ar (mm²)	117.713 ± 11.935	125.833 ± 18.137	0.142	0.427	130.400 ± 16.705	0.013*	0.039*
R_Tt.vBMD (mg HA/cm³)	344.071 ± 67.991	342.171 ± 62.805	0.776#	1.000#	380.513 ± 54.465	0.009*	0.027*
T_Tt.vBMD (mg HA/cm³)	302.288 ± 63.089	300.044 ± 48.025	0.924	1.000	347.575 ± 36.412	0.028*	0.085
R_Tb.vBMD (mg HA/cm³)	141.229 ± 39.539	159.300 ± 43.331	0.151	0.452	169.743 ± 30.688	0.008*	0.025*
T_Tb.vBMD (mg HA/cm³)	148.762 ± 47.242	158.722 ± 21.426	0.413	1.000	186.967 ± 22.081	0.002*	0.007*
R_Ct.vBMD (mg HA/cm³)	949.867 ± 42.389	926.757 ± 38.987	0.065	0.195	955.757 ± 43.695	0.437	1.000
T_Ct.vBMD (mg HA/cm³)	955.442 ± 43.572	932.178 ± 37.145	0.054#	0.161#	968.717 ± 26.908	0.856	1.000
R_Tb.BV/TV	0.205 ± 0.058	0.232 ± 0.062	0.138	0.414	0.245 ± 0.043	0.010*	0.029*
T_Tb.BV/TV	0.227 ± 0.062	0.242 ± 0.028	0.340	1.000	0.280 ± 0.028	0.001*	0.004*
R_Tb.N (1/mm)	1.325 ± 0.209	1.558 ± 0.238	0.001*	0.003*	1.637 ± 0.224	<0.001*	<0.001*
T_Tb.N (1/mm)	1.197 ± 0.209	1.309 ± 0.224	0.121#	0.363#	1.458 ± 0.222	0.001*	0.004*
R_Tb.Th (mm)	0.226 ± 0.013	0.222 ± 0.015	0.357	1.000	0.224 ± 0.013	0.474	1.000
T_Tb.Th (mm)	0.247 ± 0.020	0.247 ± 0.026	0.558#	1.000#	0.248 ± 0.011	0.760	1.000
R_Tb.Sp (mm)	0.726 ± 0.134	0.620 ± 0.119	0.007*	0.022*	0.579 ± 0.091	<0.001*	<0.001*
T_Tb.Sp (mm)	0.820 ± 0.159	0.760 ± 0.127	0.319	0.957	0.657 ± 0.099	0.003*	0.008*
R_Tb.1/N.SD (mm)	0.291 ± 0.081	0.235 ± 0.060	0.007*#	0.020*#	0.216 ± 0.042	<0.001*	<0.001*
T_Tb.1/N.SD (mm)	0.334 ± 0.101	0.327 ± 0.070	0.793#	1.000#	0.261 ± 0.044	0.003*	0.010*
R_Ct.Po (%)	0.003 ± 0.002	0.004 ± 0.002	0.091#	0.272#	0.003 ± 0.004	0.738	1.000
T_Ct.Po (%)	0.017 ± 0.011	0.016 ± 0.006	0.761#	1.000#	0.013 ± 0.005	0.373	1.000
R_Ct.Th (mm)	1.086 ± 0.155	1.086 ± 0.176	0.882#	1.000#	1.164 ± 0.159	0.093	0.279
T_Ct.Th (mm)	1.432 ± 0.196	1.444 ± 0.236	0.888	1.000	1.558 ± 0.192	0.075	0.225
R_Stiffness (N/mm)	58229.404 ± 9219.263	63859.952 ± 11530.358	0.076	0.228	65954.578 ± 10106.477	0.009*	0.026*
T_Stiffness (N/mm)	168136.258 ± 34612.992	192614.850 ± 31433.487	0.063	0.189	204437.275 ± 30526.772	0.004*	0.012*
R_Failure load (N)	3177.410 ± 516.967	3505.021 ± 635.391	0.063	0.190	3689.087 ± 568.287	0.002*	0.007*
T_Failure load (N)	9205.668 ± 1759.204	10518.231 ± 1625.399	0.051	0.154	11112.955 ± 1594.392	0.003*	0.010*

Data are presented as mean ± SD.

R_, radial; T_, tibial; Ct.Ar, cortical area; Tt.vBMD, total volumetric bone mineral density; Tb.vBMD, trabecular volumetric bone mineral density; Ct.vBMD, cortical vBMD; Tb.N, trabecular number; Tb.Th, trabecular thickness; Tb.Sp, trabecular separation; Tb.1/N.SD, inhomogeneity of trabecular network; Tb.BV/TV, trabecular bone volume fraction (bone volume/tissue volume); Ct.Th, cortical thickness; Ct.Po, cortical porosity.

For non-normally distributed variables, p-values were calculated using the Mann-Whitney U test.

*p < 0.05.

adj-p, adjusted p-value using Bonferroni correction.

#### 2.5.3 Regression analyses

To explore the relationships between bone parameters and anthropometric or biochemical factors, two separate sets of linear regression analyses were performed. First, linear regression models were fitted to examine the association between each bone parameter and BMI, with adjustment for age. Second, similar models were fitted to assess the association between each bone parameter and serum levels (e.g., inflammatory markers), adjusting for age and BMI. For each model, the regression coefficient (β), standard error, and corresponding p-value for the predictor of interest (BMI or serum parameters) were extracted, along with the coefficient of determination (R²) as a measure of model fit.

#### 2.5.4 Mediation analysis

To further explore whether the effect of group (OB-PCOS vs. OB) on bone parameters was mediated by serum testosterone levels, a mediation analysis was conducted using the ‘mediation’ package in R (29). For each bone parameter, a mediator model was first fitted using linear regression to examine the association between group and serum testosterone, followed by an outcome model that included both group and testosterone as predictors of the bone parameter. Mediation effects were estimated using the quasi-Bayesian Monte Carlo method based on the normal approximation, with 500 bootstrap simulations to generate confidence intervals and p-values for the average causal mediation effect (ACME), average direct effect (ADE), total effect, and the proportion of the total effect mediated by testosterone.

## 3 Results

The following results are presented in a hierarchical manner. We first report the primary comparisons between the two obese groups (OB-PCOS vs. OB), which were designed to isolate the skeletal effects of PCOS independent of obesity. Subsequently, we present auxiliary comparisons involving the normal-weight CON group, which serve as reference context for interpreting the direction and magnitude of bone changes associated with obesity.

### 3.1 Demographics and clinical features

The basic characteristics of the study participants are summarized in **Table 4**. The cohort comprised three groups: obese women with PCOS (OB-PCOS group, n = 23), obese women without PCOS (OB group, n = 21), and normal-weight healthy controls (CON group, n = 24). The OB-PCOS and OB groups had comparable BMI values (37.22 ± 7.19 kg/m² vs. 39.56 ± 5.95 kg/m², p = 0.132), which were significantly higher than those of the CON group (21.63 ± 2.19 kg/m², p < 0.0001). Although the OB-PCOS group was, on average, younger (29.43 ± 6.10 years) than both the OB group (33.24 ± 10.51 years) and the CON group (34.92 ± 10.71 years), there were no statistically significant differences in age among the three groups (p = 0.242). No statistically significant differences were observed in the prevalence of diabetes, hypertension, or dyslipidemia between the OB-PCOS and OB groups.

**Table 4:** Basic characteristics of the participants.

Characteristics	OB-PCOS group (n=23)	OB group (n=21)	CON group (n=24)	p-value between OB-PCOS and OB groups	p-value between three groups
Age (years)	29.43 ± 6.10	33.24 ± 10.51	34.92 ± 10.71	0.145	0.242
BMI (kg/m2)	37.22 ± 7.19	39.56 ± 5.95	21.63 ± 2.19	0.132	<0.0001*
Diabetes	7 (30.4%)	8 (38.1%)	–	0.752	–
Hypertension	9 (39.1%)	12 (57.1%)	–	0.365	–
Dyslipidiemia	2 (8.7%)	5 (23.9%)	–	0.232	–

Data are presented as mean ± SD for continuous variables, and as number (percentage) for categorical variables.

OB-PCOS, obese women with PCOS; OB, obese women without PCOS; BMI, body mass index

-, Data not applicable for CON group as they were healthy controls.

*p < 0.05

### 3.2 Clinical and biochemical characteristics of obese women with and without PCOS

Clinical and biochemical parameters were compared between the two obese groups (OB-PCOS vs. OB). The comparison of key clinical and biochemical parameters between the two groups is presented in **Table 5**. Additional parameters not shown in the main text are provided in **Supplementary Table S2**. Although most routine laboratory measurements, including complete blood count, comprehensive metabolic panel, lipid profile, oral glucose tolerance test, and thyroid function tests, were largely comparable between the groups, several parameters showed significant differences. Specifically, the OB-PCOS group exhibited significantly higher testosterone levels (p = 0.015) and cortisol levels (p = 0.013), along with elevated 3-hour post-load glucose (p = 0.019). Additionally, significant differences were observed in diastolic blood pressure (p = 0.015) between the two groups.

**Table 5:** Comparison of clinical and biochemical parameters between OB-PCOS and OB groups.

Parameters	OB-PCOS group (n=23)	OB group (n=21)	p-value
SBP (mmHg)	130.474 ± 15.939	137.875 ± 15.941	0.180
DBP (mmHg)	82.158 ± 13.442	92.750 ± 10.574	0.015*
TC (mmol/L)	4.919 ± 0.709	5.098 ± 0.790	0.432
TG (mmol/L)	1.731 ± 0.771	2.133 ± 2.180	0.972
HDL (mmol/L)	1.099 ± 0.162	1.067 ± 0.158	0.504
LDL (mmol/L)	3.221 ± 0.582	3.314 ± 0.831	0.669
FFA (μmol/L)	669.750 ± 219.503	700.167 ± 228.947	0.724
FBG (mmol/L)	6.062 ± 1.791	6.614 ± 2.466	0.821
G120 (mmol/L)	9.300 ± 4.266	8.637 ± 3.849	0.843
G180 (mmol/L)	7.356 ± 3.545	4.900 ± 1.338	0.019*
AUC_glucose	1612.147 ± 617.567	1220.684 ± 275.401	0.017*
FINS (pIU/mL)	30.891 ± 19.122	24.226 ± 14.747	0.206
I120 (pIU/mL)	162.108 ± 102.814	120.845 ± 85.862	0.221
I180 (pIU/mL)	89.837 ± 65.142	52.710 ± 49.601	0.080
AUC_insulin	23599.825 ± 11780.675	15763.167 ± 10387.413	0.059
HbA1C	5.914 ± 1.140	6.737 ± 2.208	0.329
HOMA_IR	8.070 ± 6.158	7.057 ± 4.666	0.573
IGF.1 (ng/ml)	138.688 ± 57.256	165.941 ± 59.590	0.145
LH (IU/L)	7.962 ± 6.555	10.108 ± 10.663	0.895
FSH (IU/L)	5.981 ± 2.089	9.539 ± 12.233	0.951
LH/FSH ratio	1.549 ± 1.842	1.304 ± 1.192	0.391
E2 (pg/ml)	55.609 ± 36.895	71.700 ± 50.196	0.367
Testo (ng/ml)	0.743 ± 0.372	0.502 ± 0.216	0.015*
DHEAS (μg/dl)	222.182 ± 133.128	231.556 ± 83.388	0.857
Ca (mmol/L)	2.389 ± 0.091	2.359 ± 0.091	0.271
P (mmol/L)	1.170 ± 0.136	1.178 ± 0.127	0.839
T-25OHD	14.905 ± 5.539	12.480 ± 5.399	0.076
hsCRP (mg/L)	6.427 ± 6.666	6.486 ± 3.928	0.408
IL-6 (pg/ml)	5.192 ± 9.874	3.800 ± 2.625	0.209
TNFα (pg/ml)	6.975 ± 2.086	9.775 ± 5.547	0.137

Data are presented as mean ± SD.

SBP, systolic blood pressure; DBP, diastolic blood pressure; BUN, Blood Urea Nitrogen; UA, Uric Acid; TC, Total Cholesterol; TG, Triglycerides; HDL, High-Density Lipoprotein Cholesterol; LDL, Low-Density Lipoprotein Cholesterol; FFA, Free Fatty Acids; FBG, Fasting Blood Glucose; OGTT, Oral Glucose Tolerance Test; G120, Glucose (OGTT at 120 minutes); G180, Glucose (OGTT at 180 minutes); AUC_glucose, Area Under the OGTT glucose-time Curve; FINS, Fasting Insulin; I120, Insulin (OGTT at 120 minutes); I180, Insulin (OGTT at 180 minutes); AUC_insulin, Area Under the OGTT insulin-time Curve; HbA1C, Hemoglobin A1c/Glycated Hemoglobin; HOMA_IR, Homeostatic Model Assessment for Insulin Resistance; IGF.1, Insulin-like Growth Factor 1; LH, Luteinizing Hormone; FSH, Follicle-Stimulating Hormone; E2, Estradiol; Testo, Testosterone; DHEAS, Dehydroepiandrosterone Sulfate; Ca, Calcium; P, Phosphorus; T-25OHD, Total 25-Hydroxyvitamin D; hsCRP, High-Sensitivity C-Reactive Protein; IL-6, Interleukin 6; TNFα, Tumor Necrosis Factor α.

For non-normally distributed variables, p-values were calculated using the Mann-Whitney U test.

*p < 0.05.

### 3.3 Bone strength and bone microarchitecture in obese women with and without PCOS

Detailed comparisons of bone parameters assessed by HR-pQCT between the two obese groups (OB-PCOS vs. OB) are presented in **Table 1** (distal radius) and **Table 2** (distal tibia). Overall, the OB-PCOS group exhibited significantly higher volumetric bone mineral density (vBMD) at both skeletal sites, along with specific differences in cortical and trabecular microarchitecture.

Specifically, the OB-PCOS group exhibited significantly higher total volumetric BMD at both the radius (Tt.vBMD, p = 0.036) and tibia (Tt.vBMD, p = 0.018). At the tibial site, trabecular vBMD (Tb.vBMD, p = 0.008) and trabecular bone volume fraction (Tb.BV/TV, p = 0.008) were also significantly increased in the OB-PCOS group. Furthermore, cortical vBMD was significantly higher in the OB-PCOS group at both sites (Ct.vBMD: radius, p = 0.008; tibia, p = 0.017). This was accompanied by a smaller average cortical pore diameter (Ct.Po.Dm, p = 0.023) and decreased cortical porosity (Ct.Po, p = 0.037) at the radius.

Additional differences between the two obese groups were observed in several other parameters, the OB-PCOS group showed a smaller trabecular area at the radius (Tb.Ar, p = 0.047), a smaller cortical perimeter at the radius (Ct.Pm, p = 0.037), and lower trabecular network inhomogeneity at the tibia (Tb.1/N.SD, p = 0.015). In contrast, most geometric parameters (e.g., total area, cortical area, trabecular number, trabecular thickness, and trabecular separation) and the estimated bone strength indices (stiffness and failure load) did not differ significantly between the two obese groups.

In terms of whole-bone mechanical properties estimated by finite element analysis, no significant differences were observed between the two groups. Bone stiffness, which reflects the ability of bone to resist deformation under load, and failure load, an estimate of the force required to cause fracture, were comparable between the OB-PCOS and OB groups at both the radius and tibia (all p > 0.05). These findings suggest that the higher volumetric BMD and microarchitectural advantages observed in the OB-PCOS group do not translate into enhanced estimated bone strength.

**Figure 1** presents representative reconstructed HR-pQCT images of distal radial bone microarchitecture from one participant in the OB group (Panel A) and one participant in the OB-PCOS group (Panel B). To ensure representativeness, participants with median values of total volumetric BMD within each group were selected for image display. As shown, the representative images are displayed for each group. Quantitative comparisons of trabecular and cortical parameters between the two groups are presented in **Table 1**.

**Figure 1:**
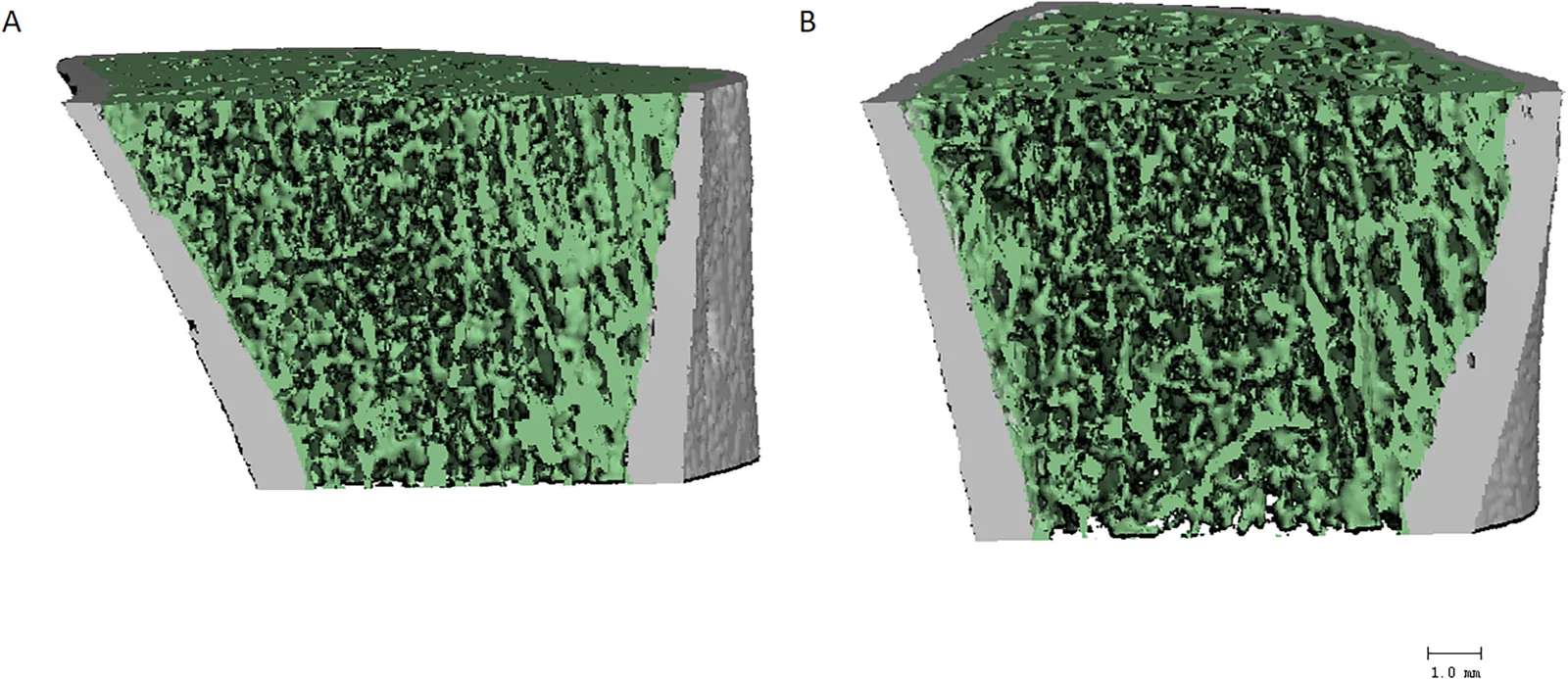
Representative reconstructed HR-pQCT images of the distal radial bone microarchitecture. Images from one participant in OB group **(A)** and one participant in OB-PCOS group **(B)**. Scale bar = 1.0 mm.

### 3.4 Comparison of bone microarchitecture and strength between obesity and normal-weight groups

As an auxiliary reference analysis, bone parameters were compared between the OB group and the normal group (**Table 3**; **Supplementary Table S3**). At the radius, the OB group exhibited significantly higher trabecular number (Tb.N, p = 0.001, adjusted p = 0.003), along with lower trabecular separation (Tb.Sp, p = 0.007, adjusted p = 0.022) and reduced trabecular network inhomogeneity (Tb.1/N.SD, p = 0.007, adjusted p = 0.020), compared with the CON group. In contrast, cortical vBMD at both the radius and tibia tended to be higher in the CON group than in the OB group, although these differences did not reach statistical significance after Bonferroni correction (radius: adjusted p = 0.195; tibia: adjusted p = 0.161). Additionally, the OB group had a larger cortical perimeter at the radius (Ct.Pm, p = 0.041); however, this difference did not remain significant after Bonferroni correction (adjusted p = 0.123). At the tibia, no significant differences were observed in any bone parameters after adjustment for multiple comparisons (all adjusted p > 0.05). Regarding estimated bone strength, although both stiffness and failure load tended to be higher in the OB group at both skeletal sites, these differences did not reach statistical significance after Bonferroni correction (all adjusted p > 0.05).

### 3.5 Comparison of bone microarchitecture and strength between OB-PCOS and CON groups

As a second auxiliary reference analysis, bone parameters were compared between the OB-PCOS group and the CON group (**Table 3**; **Supplementary Table S3**). At both the radius and tibia, the OB-PCOS group exhibited significantly higher volumetric BMD and more favorable trabecular microarchitecture compared with the normal group, with most differences remaining significant after Bonferroni correction.

Specifically, compared with the normal group, the OB-PCOS group had significantly higher total vBMD at the radius (Tt.vBMD, p = 0.009, adjusted p = 0.027), and a similar trend was observed at the tibia (Tt.vBMD, p = 0.028, adjusted p = 0.085). Trabecular vBMD was significantly higher in the OB-PCOS group at both sites (radius: Tb.vBMD, p = 0.008, adjusted p = 0.025; tibia: Tb.vBMD, p = 0.002, adjusted p = 0.007), as was trabecular bone volume fraction (radius: Tb.BV/TV, p = 0.010, adjusted p = 0.029; tibia: Tb.BV/TV, p = 0.001, adjusted p = 0.004). Regarding trabecular microarchitecture, the OB-PCOS group demonstrated significantly higher trabecular number (radius and tibia: both p < 0.001, adjusted p < 0.001 and p = 0.004, respectively), lower trabecular separation (radius: p < 0.001, adjusted p < 0.001; tibia: p = 0.003, adjusted p = 0.008), and reduced trabecular network inhomogeneity (radius: p < 0.001, adjusted p < 0.001; tibia: p = 0.003, adjusted p = 0.010). In addition, cortical area at the tibia was significantly larger in the OB-PCOS group (Ct.Ar, p = 0.013, adjusted p = 0.039), whereas no significant differences were observed in cortical vBMD, cortical porosity, or cortical thickness between the two groups at either site (all adjusted p > 0.05).

In terms of estimated bone strength, the OB-PCOS group exhibited significantly higher stiffness and failure load at both the radius (stiffness: p = 0.009, adjusted p = 0.026; failure load: p = 0.002, adjusted p = 0.007) and tibia (stiffness: p = 0.004, adjusted p = 0.012; failure load: p = 0.003, adjusted p = 0.010), with all differences remaining significant after Bonferroni correction.

### 3.6 Association between bone parameters and BMI

To further explore the impact of adiposity on skeletal parameters, we performed linear regression analyses to examine the associations of BMI with bone microarchitecture, volumetric density, and estimated bone strength, after adjusting for age. The results of these analyses are presented in **Table 6** and **Supplementary Table S4**. BMI was positively associated with estimated bone strength at the tibia, including stiffness (β = 1673.18, p = 0.014) and failure load (β = 89.28, p = 0.011). Additionally, BMI showed significant positive associations with trabecular number at both the radius (β = 0.011, p < 0.001) and tibia (β = 0.014, p = 0.003), as well as significant negative associations with trabecular separation at both sites (radius: β = -0.005, p < 0.001; tibia: β = -0.008, p = 0.006). A significant negative association was also observed between BMI and trabecular network inhomogeneity at the radius (β = -0.003, p = 0.002), but not at the tibia (p = 0.068). These findings indicate a beneficial effect of higher BMI on trabecular microarchitecture. No significant associations were detected between BMI and volumetric bone mineral density or cortical parameters at either skeletal site.

**Table 6:** Linear regression analysis of the association between BMI and bone parameters, adjusted for age.

Bone parameters	Estimate β	95%CI	p_value
R_Tb.N (1/mm)	0.011	(0.006, 0.016)	<0.001*
T_Tb.N (1/mm)	0.014	(0.005, 0.023)	0.003*
R_Tb.Sp (mm)	-0.005	(-0.007, -0.002)	<0.001*
T_Tb.Sp (mm)	-0.008	(-0.013, -0.003)	0.006*
R_Tb.1/N.SD (mm)	-0.003	(-0.004, -0.001)	0.002*
T_Stiffness (N/mm)	1673.176	(391.237, 2955.115)	0.014*
T_Failure load (N)	89.278	(23.375, 155.181)	0.011*

R_, radial; T_, tibial; Tb.N, trabecular number; Tb.Sp, trabecular separation; Tb.1/N.SD, inhomogeneity of trabecular network.

*p < 0.05.

### 3.7 Association between bone parameters and metabolic & inflammatory markers

To explore the associations of metabolic and inflammatory markers with bone parameters, linear regression analyses were performed within the OB and OB-PCOS groups (excluding the CON group), with adjustment for age and BMI. The markers examined included testosterone, DHEAS, HOMA-IR, AUC_glucose, T-25OHD, hsCRP, IL-6, and TNFα. The significant findings from these analyses are presented in **Table 7**.

**Table 7:** Linear regression analysis of the association between bone parameters and metabolic & inflammatory markers, adjusted for age and BMI.

Association	Estimate β	95%CI	p_value
R_Tt.Ar ~ AUC_glucose	-0.029	(-0.052, -0.005)	0.019*
R_Tt.Ar ~ Testo	-38.937	(-72.582, -5.292)	0.024*
R_Tb.Ar ~ AUC_glucose	-0.027	(-0.052, -0.002)	0.033*
R_Tb.Ar ~ TNFα	3.004	(0.053, 5.954)	0.046*
R_Ct.vBMD ~ TNFα	-6.155	(-9.152, -3.158)	<0.001*
R_Tb.Th ~ T.25OHD	0.001	(0.000, 0.002)	0.038*
R_Ct.Pm ~ AUC_glucose	-0.005	(-0.008, -0.001)	0.013*
R_Ct.Pm ~ TNFα	0.479	(0.055, 0.904)	0.028*
R_Ct.Pm ~ Testo	-7.118	(-12.078, -2.157)	0.006*

T-25OHD, total 25-hydroxyvitamin D; TNFα, tumor necrosis factor α; Tt.Ar, total area; Tb.Ar, trabecular area; Ct.vBMD, cortical volumetric bone mineral density; Tb.Th, trabecular thickness; Ct.Pm, cortical perimeter.

*p < 0.05.

As shown in **Table 7**, glucose metabolism-related markers showed significant associations with bone parameters. Specifically, AUC_glucose was negatively associated with total area (Tt.Ar, β = -0.029, p = 0.019), trabecular area (Tb.Ar, β = -0.027, p = 0.033), and cortical perimeter (Ct.Pm, β = -0.005, p = 0.013) at the radius.

Regarding inflammatory markers, TNFα demonstrated significant associations with several bone parameters at the radius. TNFα was positively associated with trabecular area (Tb.Ar, β = 3.004, p = 0.046) and cortical perimeter (Ct.Pm, β = 0.479, p = 0.028), but negatively associated with cortical vBMD (Ct.vBMD, β = -6.155, p < 0.001).

In terms of hormonal markers, testosterone showed significant negative associations with total area (Tt.Ar, β = -38.937, p = 0.024) and cortical perimeter (Ct.Pm, β = -7.118, p = 0.006) at the radius. Additionally, vitamin D levels (T-25OHD) were positively associated with trabecular thickness at the radius (Tb.Th, β = 0.001, p = 0.038).

### 3.8 Mediation analysis of testosterone in the association between PCOS and bone parameters

A mediation analysis was conducted to examine whether serum testosterone mediated the observed differences in bone parameters between the OB-PCOS and OB groups. The complete results of the mediation analysis are presented in **Table 8** and **Supplementary Table S5**. Overall, no statistically significant mediation effects were detected for any of the bone parameters examined. The average causal mediation effects (ACME) for all parameters yielded p-values greater than 0.05, indicating no evidence that testosterone mediated the group-related differences in bone microarchitecture, volumetric bone density, or estimated bone strength at either skeletal site.

**Table 8:** Mediation analysis of testosterone on the association between group (OB-PCOS vs. OB) and bone parameters.

Parameter	ACME	ACME_p	ADE	ADE_p	Total_Effect	Total_p
R_Tb.Ar (mm²)	-5.14	0.432	-15.43	0.212	-20.56	0.048*
R_Tt.vBMD (mg HA/cm³)	4.23	0.692	33.35	0.108	37.58	0.036*
T_Tt.vBMD (mg HA/cm³)	-2.08	0.600	54.07	0.004*	51.99	0.004*
T_Tb.vBMD (mg HA/cm³)	-1.35	0.448	30.97	0.008*	29.62	0.008*
R_Ct.vBMD (mg HA/cm³)	0.86	0.948	24.36	0.100	25.22	0.048*
T_Ct.vBMD (mg HA/cm³)	-3.18	0.304	41.7	0.004*	38.52	0.004*
T_Tb.BV/TV	0.00	0.600	0.04	0.004*	0.04	0.004*
T_Tb.Sp (mm)	0.01	0.504	-0.11	0.040*	-0.1	0.052
T_Tb.1/N.SD (mm)	0.00	0.500	-0.07	0.028*	-0.06	0.032*
R_Ct.Pm (mm)	-1.21	0.304	-2.08	0.264	-3.29	0.048*
R_Ct.Po.Dm (mm)	0.00	0.148	-0.01	0.088	-0.01	0.008*

ACME, average causal mediation effect; ADE, average direct effect; Tb.Ar, trabecular area; Tt.vBMD, total volumetric bone mineral density; Tb.vBMD, trabecular volumetric bone mineral density; Ct.vBMD, cortical vBMD; Tb.Sp, trabecular separation; Tb.1/N.SD, inhomogeneity of trabecular network; Ct.Pm, cortical perimeter; Ct.Po.DM, cortical pore diameter.

Although the mediation effects were not significant, several bone parameters exhibited significant average direct effects (ADE), indicating that group membership (OB-PCOS vs. OB) independently influenced these parameters after accounting for testosterone levels. Specifically, significant direct effects were observed for tibial total vBMD (Tt.vBMD, p = 0.004), tibial trabecular vBMD (Tb.vBMD, p = 0.008), tibial cortical vBMD (Ct.vBMD, p = 0.004), tibial trabecular bone volume fraction (Tb.BV/TV, p = 0.004), tibial trabecular network inhomogeneity (Tb.1/N.SD, p = 0.028), and tibial trabecular separation (Tb.Sp, p = 0.040). These findings suggest that while no evidence of mediation by testosterone was observed, the direct effect of OB-PCOS status on these tibial parameters remains significant even after adjustment for testosterone levels.

## 4 Discussion

This HR-pQCT study systematically evaluated the effects of PCOS and obesity on bone microarchitecture and estimated bone strength by comparing obese women with PCOS, obese women without PCOS, and normal-weight controls. Our findings demonstrate that although PCOS is associated with higher vBMD compared with both obese and normal-weight controls, this advantage does not translate into enhanced estimated bone strength. Furthermore, testosterone levels do not mediate the PCOS-related skeletal differences, while higher BMI is significantly associated with improved trabecular microarchitecture and increased estimated bone strength in numerical terms.

In the present study, compared with BMI-matched obese women without PCOS, obese women with PCOS exhibited significantly higher total vBMD (Tt.vBMD) and cortical vBMD (Ct.vBMD) at both the radius and tibia, as well as significantly higher trabecular vBMD (Tb.vBMD) and trabecular bone volume fraction (Tb.BV/TV) at the tibial site. This observation is corroborated by Kassanos et al. (30), who found increased cortical bone density in women with PCOS.

Given the well-established anabolic effects of androgens on bone (31), we hypothesized that the higher bone mineral density observed in the OB-PCOS group might be attributable to elevated testosterone levels. Indeed, the OB-PCOS group exhibited significantly higher testosterone concentrations. However, mediation analysis revealed that testosterone did not significantly mediate the association between PCOS and bone parameters, whereas PCOS status itself had significant direct effects on multiple tibial bone parameters (e.g., Tt.vBMD, Tb.vBMD, Ct.vBMD). It should be noted that the serum testosterone measured in this study represents total testosterone, which may not fully reflect the biologically active free fraction. Given that PCOS is often associated with reduced sex hormone-binding globulin (SHBG) levels (32), free testosterone indices might provide a more accurate assessment of androgen activity. Nevertheless, within the limitations of total testosterone measurement, our findings suggest that the PCOS-related bone phenotype may be driven by other characteristics of the syndrome itself rather than by hyperandrogenemia alone.

Notably, despite their higher bone density, the OB-PCOS group did not demonstrate corresponding improvements in estimated bone strength (stiffness and failure load). This decoupling between bone density and bone strength suggests that PCOS-related metabolic alterations may affect not only volumetric bone mineral density but also the material properties of bone tissue. Studies have shown that patients with PCOS often present with low-grade chronic inflammation (33), and inflammatory cytokines may impair bone material properties by affecting collagen cross-linking (34). Together, these findings point to the possibility that PCOS-associated metabolic disturbances (such as insulin resistance or chronic inflammation) may exert dual effects: contributing to the observed bone density phenotype while simultaneously compromising bone material quality, thereby limiting the expected gain in bone strength.

To further explore the mechanisms underlying the dissociation between bone density and strength, we examined associations of metabolic and inflammatory markers with bone parameters within the OB and OB-PCOS groups. Notably, TNFα exhibited a divergent association pattern: higher TNFα was associated with larger trabecular area and cortical perimeter, yet was strongly negatively associated with cortical vBMD. This dual pattern suggests that chronic inflammation may promote geometric expansion while simultaneously impairing cortical mineralization—a combination that could underlie the observed density–strength decoupling and partly explain why higher vBMD in the OB-PCOS group did not translate into superior estimated bone strength. In contrast, post-challenge glucose (AUC_glucose) was inversely associated with total area, trabecular area, and cortical perimeter at the radius, indicating that impaired glucose tolerance may constrain skeletal geometric expansion. Testosterone also showed inverse associations with total area and cortical perimeter, further supporting our mediation finding that hyperandrogenemia does not drive the PCOS-related bone phenotype. Vitamin D was positively associated with trabecular thickness, consistent with its known role in bone mineralization.

Obesity is known to exert a beneficial effect on bone, primarily through mechanical loading (16). In the present study, higher BMI was significantly associated with improved trabecular microarchitecture (e.g., higher Tb.N, lower Tb.Sp) at the radius and tended to be associated with higher estimated bone strength at both skeletal sites. Notably, these favorable trabecular associations were observed predominantly at the radius, whereas no significant differences were detected at the tibia after multiple comparison correction. This regional discrepancy may reflect the higher trabecular-to-cortical ratio and greater metabolic activity of the radius, as well as the possibility that the tibia, being a major weight-bearing bone, may already be maximally adapted to mechanical loading, thereby masking additional obesity-related effects at this site. In addition to mechanical loading, leptin and other adiposity-related factors may also influence bone metabolism, as demonstrated in rodent studies (35). However, these findings cannot be directly extrapolated to human obesity, which is typically characterized by hyperleptinemia and leptin resistance rather than leptin deficiency. Given the complex and context-dependent skeletal effects of leptin, its net contribution to the favorable bone phenotype in human obesity remains uncertain, and it may act in concert with mechanical loading and other adiposity-related factors (16).

This study has several limitations. First, the cross-sectional design precludes causal inferences; longitudinal studies are warranted to establish whether the observed skeletal differences translate into differential fracture risk over time. Second, the sample size for the tibial site was relatively small (OB-PCOS, n = 12; OB, n = 9), as tibial HR-pQCT scans were performed only in a subset of participants; scans at the tibia were not feasible for some participants due to anthropometric limitations, as their lower limb circumference exceeded the scanner’s acquisition space. No formal sample size calculation was performed prior to enrollment, as this was an exploratory cross-sectional study. Based on the observed variances in tibial stiffness, our sample would have been adequately powered to detect only relatively large effect sizes (Cohen’s d ≥ ~1.0), corresponding to approximately a 15–16% difference in estimated bone stiffness between groups, whereas the actual observed difference was only about 6% and was not statistically significant. Therefore, smaller but potentially clinically meaningful differences in estimated bone strength could have been missed due to limited statistical power. This increases the risk of type II error; larger cohorts with complete tibial assessments are needed to confirm these site-specific observations. Third, the absence of bone turnover marker data prevents comprehensive assessment of bone metabolic dynamics; future studies incorporating such markers (such as β-CTX and PINP) would help elucidate underlying mechanisms. Fourth, lifestyle factors (e.g., smoking, alcohol consumption, physical activity, and education level) and reproductive characteristics (e.g., age at menarche, parity, and menopausal status) were not systematically collected in this study, as the primary focus was on HR-pQCT-derived bone parameters and these variables were not part of our routine clinical data collection at the time of enrollment. Although the OB-PCOS and OB groups were matched for age and BMI, and we adjusted for these variables in regression analyses, we cannot completely exclude the possibility of residual confounding due to these unmeasured factors. Future studies should incorporate comprehensive assessments of these variables to better delineate their contributions to the skeletal differences observed between obese women with and without PCOS. In addition, bone strength estimated by finite element analysis serves as a surrogate indicator and does not directly reflect actual fracture risk. Prospective studies with fracture endpoints are ultimately required to determine the clinical significance of our findings. Finally, the measurement of total rather than free testosterone may not fully capture biologically active androgen fractions. Future investigations should incorporate free testosterone or calculated free androgen indices.

In conclusion, this study demonstrates that in obese women with PCOS, higher volumetric BMD and more favorable trabecular microarchitecture do not translate into enhanced estimated bone strength compared with BMI-matched obese women without PCOS. Notably, cortical vBMD at both the radius and tibia tended to be lower in obese individuals than in normal-weight controls, although these differences did not reach statistical significance after correction, suggesting that the overall BMD advantage in obesity is predominantly driven by trabecular rather than cortical bone. This density-strength discordance, together with the finding that we observed no evidence of testosterone mediating the PCOS-related bone differences, suggests that PCOS exerts effects on bone that extend beyond hyperandrogenemia and standard microarchitectural measures, likely involving alterations in bone material properties. Furthermore, the beneficial trabecular effects of obesity were observed primarily at the radius, highlighting the site-specific nature of the skeletal response to obesity. In contrast, obesity itself remains positively associated with favorable trabecular microarchitecture; however, this did not translate into significantly enhanced estimated bone strength in our study population, and cortical vBMD even showed a trend toward lower values in obese individuals. Prospective studies are warranted to determine the clinical significance of these structural and material-level alterations for fracture risk.

## Data Availability

The raw data supporting the conclusions of this article will be made available by the authors, without undue reservation.
