# Supplementary material for: Bone microarchitecture and strength in obese women with and without polycystic ovary syndrome (PCOS): a peripheral quantitative computed tomography (pQCT) study
Source: Front Endocrinol (Lausanne). 2026 Sep 25;17:1935187. doi: 10.3389/fendo.2026.1935187 (PMC13649439; doi:10.3389/fendo.2026.1935187)
Supplement: Supplementary file 1 [file SupplementaryFile1.docx]

**Appendix for**

**Bone Microstructure and Strength in Obese Women With and Without Polycystic Ovary Syndrome (PCOS): A Peripheral Quantitative Computed Tomography (pQCT) Study**

This file includes:

**Table S1**. Summary of biochemical assay kits used in this study.

**Table S2 (related to Table 2).** Additional clinical and biochemical parameters in OB-PCOS and OB groups.

**Table S3 (related to Table 5).** Additional comparison of bone microarchitecture and estimated strength between OB, OB-PCOS and CON group.

**Table S4 (related to Table 6).** Additional Linear regression analysis of the association between BMI and bone parameters, adjusted for age.

**Table S5 (related to Table 8).** Additional Mediation analysis of testosterone on the association between group (OB-PCOS vs. OB) and bone parameters.

**Figure S1.** Box Plots of Age / BMI across Three Groups.

**Figure S2.** Box Plots of Age / BMI across Obese and Normal Groups.

**Table S1.** Summary of biochemical assay kits used in this study.

| Parameter | Assay Method | Instrument / Platform | Kit Name / Reagent | Manufacturer |
| --- | --- | --- | --- | --- |
| IGF-1 | Chemiluminescent enzyme immunometric assay | Immulite 2000 | Immulite 2000 IGF-1 Kit | Siemens Healthcare Diagnostics |
| TSH | CLIA | Beckman DXI800 | Beckman Access TSH Kit | Beckman |
| FT3 | CLIA | Siemens Atellica | Atellica FT3 Kit | Siemens Healthcare Diagnostics |
| FT4 |  |  | Atellica FT4 Kit |  |
| Glucose | Enzymatic method | Cobas c702 (Roche) | NA | Roche Diagnostics (Shanghai, China) |
| Insulin | CLIA | NA | NA | NA |
| ACTH | CLIA | Advia Centaur XP | NA | Siemens Healthcare Diagnostics |
| Cortisol |  | Siemens Atellica | Atellica Cortisol Kit |  |
| LH | CLIA | ACS: 180 Automatic Chemiluminescence System | NA | Siemens Healthcare Diagnostics |
| FSH |  |  |  |  |
| PRL |  |  |  |  |
| Estradiol |  |  |  |  |
| Progesterone |  |  |  |  |
| Testosterone |  |  |  |  |

Abbreviations: CLIA, Chemiluminescence immunoassay; NA, not available.

**Table S2 (related to Table 2).** Additional clinical and biochemical parameters in OB-PCOS and OB groups.

|  | **OB-PCOS group (n=23)** | **OB group (n=21)** | p-value |
| --- | --- | --- | --- |
| CR (μmol/L) | 63.130 ± 9.488 | 58.095 ± 8.342 | 0.070 |
| Apoa1 (g/L) | 1.278 ± 0.159 | 1.282 ± 0.178 | 0.948 |
| Apob (g/L) | 0.998 ± 0.163 | 1.022 ± 0.156 | 0.704 |
| LP(a) (mg/L) | 146.688 ± 203.026 | 117.250 ± 215.946 | 0.378 |
| GH (ng/ml) | 0.364 ± 0.636 | 0.285 ± 0.208 | 0.452 |
| Prog (ng/ml) | 0.610 ± 0.607 | 2.443 ± 4.274 | 0.622 |
| PRL (ng/ml) | 17.662 ± 12.058 | 13.040 ± 5.487 | 0.273 |

Abbreviations: CR, Creatinine; Apoa1, Apolipoprotein A1; Apob, Apolipoprotein B; LP(a), Lipoprotein (a); GH, Growth Hormone; Prog, Progesterone; PRL, Prolactin.

For non-normally distributed variables, p-values were calculated using the Mann-Whitney U test.

**Table S3 (related to Table 5).** Additional comparison of bone microarchitecture and estimated strength between OB, OB-PCOS and CON group.

| Parameter | CON group (n=24) | OB group (n=21) | p_value (OB vs. CON) | adj_p (OB vs. CON) | OB-PCOS group (n=23) | p_value (OB-PCOS vs. CON) | adj_p (OB-PCOS vs. CON) |
| --- | --- | --- | --- | --- | --- | --- | --- |
| R_Tt.Ar (mm²) | 230.983 ± 47.676 | 248.690 ± 26.462 | 0.126 | 0.379 | 229.261 ± 40.167 | 0.894 | 1.000 |
| T_Tt.Ar (mm²) | 618.133 ± 110.209 | 690.456 ± 118.191 | 0.110 | 0.329 | 628.725 ± 68.847 | 0.763 | 1.000 |
| R_Tb.Ar (mm²) | 176.608 ± 45.549 | 193.205 ± 29.050 | 0.159 | 0.477 | 171.683 ± 39.378 | 0.694 | 1.000 |
| T_Tb.Ar (mm²) | 505.429 ± 109.372 | 569.944 ± 120.312 | 0.152 | 0.455 | 503.367 ± 65.823 | 0.953 | 1.000 |
| R_Ct.Pm (mm) | 62.663 ± 6.665 | 66.038 ± 3.802 | 0.041* | 0.123 | 62.600 ± 6.323 | 0.974 | 1.000 |
| T_Ct.Pm (mm) | 95.992 ± 8.670 | 102.100 ± 8.300 | 0.078 | 0.234 | 96.967 ± 5.483 | 0.725 | 1.000 |
| R_Ct.Po.Dm (mm) | 0.141 ± 0.027 | 0.153 ± 0.023 | 0.102 | 0.306 | 0.138 ± 0.020 | 0.700 | 1.000 |
| T_Ct.Po.Dm (mm) | 0.218 ± 0.026 | 0.220 ± 0.029 | 0.921 | 1.000 | 0.208 ± 0.037 | 0.341 | 1.000 |

Data are presented as mean ± SD.

Abbreviations: R_, radial; T_, tibial; Tt.Ar, total area; Tb.Ar, trabecular area; Ct.Pm, cortical perimeter; Ct.Po.DM, cortical pore diameter.

*: p < 0.05.

adj-p: adjusted p-value using Bonferroni correction.

**Table S4 (related to Table 6).** Additional Linear regression analysis of the association between BMI and bone parameters, adjusted for age.

|  | Estimate β | 95%CI | p_value |
| --- | --- | --- | --- |
| R_Tt.Ar (mm²) | 0.504 | (-0.484, 1.491) | 0.321 |
| T_Tt.Ar (mm²) | 3.101 | (-1.247, 7.449) | 0.169 |
| R_Tb.Ar (mm²) | 0.434 | (-0.546, 1.414) | 0.389 |
| T_Tb.Ar (mm²) | 2.543 | (-1.768, 6.855) | 0.254 |
| R_Ct.Ar (mm²) | 0.148 | (-0.034, 0.330) | 0.116 |
| T_Ct.Ar (mm²) | 0.572 | (-0.034, 1.177) | 0.071 |
| R_Tt.vBMD (mg HA/cm³) | 0.373 | (-1.198, 1.945) | 0.643 |
| T_Tt.vBMD (mg HA/cm³) | 0.955 | (-1.148, 3.057) | 0.378 |
| R_Tb.vBMD (mg HA/cm³) | 0.676 | (-0.233, 1.586) | 0.150 |
| T_Tb.vBMD (mg HA/cm³) | 1.383 | (-0.040, 2.805) | 0.064 |
| R_Ct.vBMD (mg HA/cm³) | -0.608 | (-1.664, 0.448) | 0.263 |
| T_Ct.vBMD (mg HA/cm³) | -0.839 | (-2.372, 0.694) | 0.289 |
| R_Tb.BV/TV | 0.001 | (-0.000, 0.002) | 0.140 |
| T_Tb.BV/TV | 0.002 | (0.000, 0.004) | 0.053 |
| R_Tb.Th (mm) | 0 | (-0.001, 0.000) | 0.180 |
| T_Tb.Th (mm) | 0 | (-0.001, 0.001) | 0.796 |
| T_Tb.1/N.SD (mm) | -0.003 | (-0.006, 0.000) | 0.068 |
| R_Ct.Pm (mm) | 0.134 | (-0.011, 0.278) | 0.075 |
| T_Ct.Pm (mm) | 0.268 | (-0.065, 0.602) | 0.123 |
| R_Ct.Po (%) | 0 | (-0.000, 0.000) | 0.078 |
| T_Ct.Po (%) | 0 | (-0.000, 0.000) | 0.542 |
| R_Ct.Th (mm) | 0.002 | (-0.002, 0.006) | 0.403 |
| T_Ct.Th (mm) | 0.003 | (-0.005, 0.011) | 0.504 |
| R_Ct.Po.Dm (mm) | 0 | (-0.000, 0.001) | 0.105 |
| T_Ct.Po.Dm (mm) | 0 | (-0.002, 0.001) | 0.565 |
| R_Stiffness (N/mm) | 212.075 | (-43.626, 467.776) | 0.109 |
| R_Failure load (N) | 13.984 | (-0.336, 28.304) | 0.060 |

Abbreviations: R_, radial; T_, tibial; Tt.Ar, total area; Tb.Ar, trabecular area; Ct.Ar, cortical area; Tt.vBMD, total volumetric bone mineral density; Tb.vBMD, trabecular volumetric bone mineral density; Ct.vBMD, cortical vBMD; Tb.Th, trabecular thickness; Tb.1/N.SD, inhomogeneity of trabecular network; Tb.BV/TV, trabecular bone volume fraction (bone volume/tissue volume); Ct.Pm, cortical perimeter; Ct.Th, cortical thickness; Ct.Po, cortical porosity; Ct.Po.DM, cortical pore diameter.

**Table S5 (related to Table 8).** Additional Mediation analysis of testosterone on the association between group (OB-PCOS vs. OB) and bone parameters.

| Parameter | ACME | ACME_p | ADE | ADE_p | Total_Effect | Total_p |
| --- | --- | --- | --- | --- | --- | --- |
| R_Tt.Ar (mm²) | -5.35 | 0.492 | -13.24 | 0.236 | -18.58 | 0.072 |
| T_Tt.Ar (mm²) | -5.45 | 0.408 | -60.18 | 0.232 | -65.62 | 0.148 |
| T_Tb.Ar (mm²) | -4.02 | 0.488 | -68.65 | 0.172 | -72.67 | 0.096 |
| R_Ct.Ar (mm²) | -0.62 | 0.620 | 1.65 | 0.568 | 1.03 | 0.692 |
| T_Ct.Ar (mm²) | -1.46 | 0.288 | 8.21 | 0.308 | 6.75 | 0.376 |
| R_Tb.vBMD (mg HA/cm³) | 1.33 | 0.828 | 10.62 | 0.376 | 11.95 | 0.328 |
| R_Tb.BV/TV | 0.00 | 0.696 | 0.01 | 0.392 | 0.02 | 0.288 |
| R_Tb.N (1/mm) | 0.00 | 0.832 | 0.08 | 0.332 | 0.08 | 0.244 |
| T_Tb.N (1/mm) | -0.02 | 0.412 | 0.16 | 0.188 | 0.14 | 0.180 |
| R_Tb.Th (mm) | 0.00 | 0.628 | 0.00 | 0.456 | 0.00 | 0.544 |
| T_Tb.Th (mm) | 0.00 | 0.764 | 0.00 | 0.836 | 0.00 | 0.892 |
| R_Tb.Sp (mm) | -0.01 | 0.612 | -0.04 | 0.360 | -0.04 | 0.232 |
| R_Tb.1/N.SD (mm) | 0.00 | 0.856 | -0.02 | 0.288 | -0.02 | 0.204 |
| T_Ct.Pm (mm) | -0.47 | 0.380 | -4.75 | 0.196 | -5.22 | 0.108 |
| R_Ct.Po (%) | 0.00 | 0.076 | 0.00 | 0.928 | 0.00 | 0.536 |
| T_Ct.Po (%) | 0.00 | 0.936 | 0.00 | 0.304 | 0.00 | 0.300 |
| R_Ct.Th (mm) | 0.00 | 0.864 | 0.07 | 0.228 | 0.07 | 0.144 |
| T_Ct.Th (mm) | -0.01 | 0.760 | 0.15 | 0.160 | 0.14 | 0.176 |
| T_Ct.Po.Dm (mm) | 0.00 | 0.808 | -0.01 | 0.576 | -0.01 | 0.500 |
| R_Stiffness (N/mm) | -831 | 0.628 | 2933.5 | 0.476 | 2102.5 | 0.600 |
| T_Stiffness (N/mm) | -1679.02 | 0.344 | 21759.15 | 0.140 | 20080.13 | 0.168 |
| R_Failure load (N) | -44.72 | 0.700 | 228.64 | 0.276 | 183.92 | 0.312 |
| T_Failure load (N) | -92.47 | 0.280 | 1118.09 | 0.088 | 1025.62 | 0.104 |

Abbreviations: ACME, average causal mediation effect; ADE, average direct effect; Tt.Ar, total area; Tb.Ar, trabecular area; Ct.Ar, cortical area; Tb.vBMD, trabecular volumetric bone mineral density; Tb.N, trabecular number; Tb.Th, trabecular thickness; Tb.Sp, trabecular separation; Tb.1/N.SD, inhomogeneity of trabecular network; Tb.BV/TV, trabecular bone volume fraction (bone volume/tissue volume); Ct.Pm, cortical perimeter; Ct.Th, cortical thickness; Ct.Po, cortical porosity; CT.Po.DM, cortical pore diameter.

**Figure S1.** Box Plots of Age / BMI across Three Groups.

Kruskal-Wallis test & Dunn's post hoc test; ****: p<0.0001

**Figure S2.** Box Plots of Age / BMI across Obese and Normal Groups.

****: p<0.0001
